# 4209 Early Electrographic Seizure Detection by Neuro ICU Nurses via Bedside Real-Time Quantitative EEG

**DOI:** 10.1017/cts.2020.124

**Published:** 2020-07-29

**Authors:** Safa Kaleem, Jennifer H. Kang, Alok Sahgal, Christian E. Hernandez, Christa B. Swisher

**Affiliations:** 1 Duke University

## Abstract

OBJECTIVES/GOALS:

1. Determine test characteristics of Neuro ICU nurse interpretation of real-time bedside qEEG for seizure detection
2. Determine difference in time to detection of seizures between qEEG interpretation and raw cEEG reads
3. Determine how seizure characteristics affect accuracy of qEEG reads

METHODS/STUDY POPULATION:

- Subjects: Nurses caring for patients admitted to the Neuro ICU at Duke University Hospital who are initiated on cEEG.
- Nurses evaluate qEEG display at the bedside on an hourly basis after undergoing a standardized qEEG training session. The standard practice of independent review of cEEG and treatment by the Neuro ICU team remains unchanged.
- Post-hoc review of cEEG data by two blinded, board-certified neurophysiologists will be performed for each patient. The raw cEEG data will be scored for the number of seizures present per hour, background, seizure duration, and seizure spatial extent.
- The time from first seizure occurrence to clinical recognition will be recorded.

RESULTS/ANTICIPATED RESULTS:

- Thus far, 91 patients with 583 1-hour blocks of nurse interpretations have been studied, with 6 patients experiencing seizures while on study. Enrollment will be completed on 1/17/20
- Preliminary data show a sensitivity of 95.8% (79.9%, 99.9%), specificity of 95.2 (93.1%, 96.8%), positive predictive value of 46.0% (36.9%, 55.4%), negative predictive value of 99.8% (98.7%, 99.9%), positive likelihood ratio of 19.8 (13.6, 28.9), negative likelihood ratio (0.04 (0.01, 0.3). All confidence intervals are 95%. False alarm rate is 0.05/hour.
- Further analyses are pending completion of enrollment in January 2020.

DISCUSSION/SIGNIFICANCE OF IMPACT: Nurse interpretation of real-time bedside qEEG for seizure detection is feasible in the Duke Neuro ICU. QEEG functions well as a screening tool with good specificity and low false alarm rate. Use of qEEG by nurses could lead to shorter time to seizure detection, which may improve patient outcomes. CONFLICT OF INTEREST DESCRIPTION: Safa Kaleem, BS: Research reported in this publication was supported by a Pfizer Foundation grant and the Duke Clinical Translational Science Institute (CTSI). The content is solely the responsibility of the authors and does not necessarily represent the official views of the Pfizer Foundation or the Duke CTSI. Jennifer H. Kang, MD: None to declare. Alok Sahgal, MD: None to declare. Christa B. Swisher, MD: Received speaker’s honorarium from EISAI and UCB.

